# Rationalization of the X-ray photoelectron spectroscopy of aluminium phosphates synthesized from different precursors†

**DOI:** 10.1039/c9ra08738a

**Published:** 2020-02-26

**Authors:** Victoria Bemmer, Michael Bowker, James H. Carter, Philip R. Davies, Lee E. Edwards, Kenneth D. M. Harris, Colan E. Hughes, Fiona Robinson, David J. Morgan, Matthew G. Thomas

**Affiliations:** Cardiff Catalysis Institute, School of Chemistry, Cardiff University Cardiff CF10 3AT UK daviespr@cardiff.ac.uk; School of Chemistry, Cardiff University Cardiff CF10 3AT UK; Cogent Power Ltd Newport NP19 0RB UK; Dept. of Materials, Imperial College, South Kensington Campus London SW7 2AZ UK

## Abstract

The aim of this paper is to clarify the assignments of X-ray photoelectron spectra of aluminium phosphate materials prepared from the reaction of phosphoric acid with three different aluminium precursors [Al(OH)_3_, Al(NO_3_)_3_ and AlCl_3_] at different annealing temperatures. The materials prepared have been studied by X-ray photoelectron spectroscopy (XPS), powder X-ray diffraction (XRD), infrared spectroscopy and high-resolution solid-state ^31^P NMR spectroscopy. A progressive polymerization from orthophosphate to metaphosphates is observed by XRD, ATR-FTIR and solid state ^31^P NMR, and on this basis the oxygen states observed in the XP spectra at 532.3 eV and 533.7 eV are assigned to P–O–Al and P–O–P environments, respectively. The presence of cyclic polyphosphates at the surface of the samples is also evident.

## Abstract 2

Using NMR, XRD and FTIR we clarify the assignment of XP spectra of aluminium phosphates prepared from three different aluminium precursors [Al(OH)_3_, Al(NO_3_)_3_ and AlCl_3_] at different annealing temperatures.

## Graphical abstract

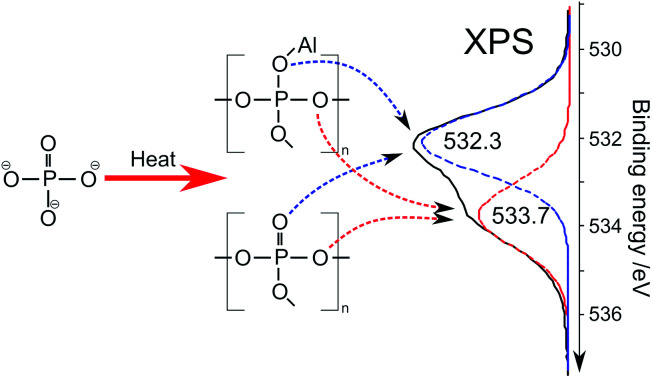

## Introduction

There are a wide array of applications of phosphate based materials, including biomedical (due to the natural occurrence of phosphates in physiology and their biocompatibility^1–4^), ceramics (due to their high strength and thermal stability) and refractory materials (in which phosphates are frequently used as binders^5^). Curing temperatures of phosphates are critical to their performance in the latter applications and as a result there has been a concerted effort to understand the thermal evolution of phosphate systems. Heating induces a progressive polymerization starting from orthophosphates (PO_4_^3−^), which polymerize to form a series of polyphosphates such as the pyrophosphates (P_2_O_7_^4−^) and finally metaphosphates, which are long range cross-linked networks (PO_3_^−^)_*n*_.^6^ This paper is focused on aluminium phosphates generated from a mixture of phosphoric acid and three different aluminium based precursors: Al(OH)_3_, Al(NO_3_)_3_ and AlCl_3_.

The majority of studies on aluminium phosphates have focused on bulk analysis techniques,^7^ such as powder X-ray diffraction (XRD), solid-state ^31^P NMR, FTIR and thermogravimetric methods, but the surface properties of these materials are also of interest and one of the most commonly used techniques to investigate this aspect is X-ray photoelectron spectroscopy (XPS). A seminal study by Gresch *et al.*^8^ in 1979 on XPS of sodium phosphates provided well substantiated peak assignments for the oxygen region. This paper has since been extensively cited and used as a benchmark for XPS studies of phosphates. Gresch *et al.* proposed that the different states of oxygen created by crosslinking between phosphate units could be distinguished by their XP spectra, with the “bridging” oxygens appearing at higher binding energy (533.1–533.6 eV) than “non-bridging” oxygens (530.5–531.7 eV). This assignment was based on electronegativity arguments and spectra of model compounds. Concomitant with the shift in O(1s) binding energy, Gresch *et al.* reported that the P(2p) binding energy shifts from 132.5 eV to 134.5 eV as the degree of P–O–P bridging bonds increases.

More recently, Crobu *et al.*^9^ have also used the shift in O(1s) binding energy to assess the ratio of bridging to non-bridging oxygen in zinc polyphosphate glasses, correlating their results with secondary ion mass spectrometry measurements. They reported a shift from 532.2 eV to 534.0 eV as the reference material changed from an orthophosphate to a metaphosphate, with the P(2p) peak shifting from 134.0 eV to 134.8 eV.

Rotole and Sherwood, on the other hand, studied electrochemically deposited phosphate films on aluminium substrates.^10^ The XP spectra were referenced against data on aluminium orthophosphate^11^ and metaphosphate^12^ samples. They reported a constant P(2p) binding energy of 134.5 eV and a shift in the O(1s) peak from 531.4 eV for the orthophosphate to a broader peak at 531.8 eV for the metaphosphate. The latter peak clearly showed evidence for a second component at about 533.5 eV. However, the O(1s) data from the electrochemically treated surface were not so conclusive; the O(1s) spectrum of the “metaphosphate” was significantly broader than that of the orthophosphate and seemed to consist of two components. It is not clear whether the component due to bridging oxygens is at higher or lower binding energy than the component due to terminal oxygens.

In the present study, we have used solid-state ^31^P NMR, powder XRD and FTIR data to explore the structural changes that occur in aluminium phosphate materials synthesized from 3 : 1 mixtures of phosphoric acid and an aluminium precursor (either aluminium hydroxide, aluminium nitrate or aluminium chloride), which throw light on the information available from the surface specific XPS technique. Our results show that the expected polymerization occurs in materials prepared from all three precursors, and largely confirm the assignments of XP spectra based on existing literature. However, we also find evidence for the presence, at the surface, of polyphosphate species that do not contain aluminium, which distorts the Al : O : P ratios established from the XPS spectra.

## Experimental

### Synthesis of aluminium phosphate powders

The aluminium precursor AlX_3_, where X = OH (Sigma-Aldrich, Reagent Grade), NO_3_ (Sigma-Aldrich, ACS Reagent, >98%) or Cl (Sigma-Aldrich, 99.99%), was added to water (50 ml) at 298 K and mixed until dissolved. H_3_PO_4_ (8.75 ml, Sigma-Aldrich, 85% wt, 99.99% purity) was added to the AlX_3_ solution to give a P : Al ratio of 3 : 1 and the solution was allowed to mix for 30 min. The reaction mixture was then heated on a hotplate to evaporate the water, resulting in the formation of a viscous gel. Separate samples of the gel were heated in air in a furnace for a period of one hour at three different temperatures (300, 500 and 800 °C), and then allowed to cool to room temperature before analysis. In the case of the materials prepared from Al(OH)_3_ and Al(NO_3_)_3_, a slightly wet white powder was obtained on heating to 300 °C which was observed (by eye) to dry completely on heating at the higher temperatures. However, the samples prepared from AlCl_3_ remained gel-like at 300 °C. These samples were stable under vacuum and so could be analysed by XPS, but they had to be heated to 400 °C to obtain a solid that was suitable for analysis by solid-state NMR and ATR could be obtained. In the discussion below, the materials prepared from the Al(OH)_3_, Al(Cl)_3_ and Al(NO_3_)_3_ precursors are labelled AlP_OH_, AlP_Cl_ and AlP_NO_3__, respectively.

### Materials characterization

XP spectra were recorded at room temperature on the powder samples using a Kratos Axis Ultra-DLD photoelectron spectrometer with a monochromatic Al Kα X-ray source in the “hybrid spectroscopy” mode resulting in an analysis area of 700 × 300 μm^2^ at a pass-energy of 40 eV for high-resolution scans and 160 eV for survey scans. The XPS data were analysed using CasaXPS^13^ with all binding energies referenced to the C(1s) peak at 284.7 eV with an uncertainty of ∼0.2 eV. Since intensities for powder samples are dependent on the surface area analysed, which is poorly reproducible between different powder samples, the XP spectra shown in the figures are normalized to the point of maximum intensity. Curve fits were made using Gaussian–Lorentzian (GL (30)) line-shapes.

Powder X-ray diffraction (XRD) data were recorded at room temperature using a PANalytical X'Pert Pro diffractometer with a monochromatic Cu Kα source (*λ* = 0.154 nm) operating at 40 kV and 40 mA. The data were recorded over the 2*θ* range 10–80° with a step size of 0.016°.

High-resolution solid-state ^31^P NMR spectra were acquired at room temperature on a Chemagnetics Infinity Plus spectrometer (^31^P Larmor frequency, 121.50 MHz). The samples were contained in a 4 mm rotor with magic-angle spinning at 12 kHz. Methyldiphenylphosphine oxide (MDPPO) was used as a reference, with ^31^P chemical shift at 30.8 ppm.

FTIR spectra were recorded using a germanium crystal ATR on a Varian 3100 Excalibur system with Varian Resolutions Pro software.

## Results

### Bulk structural analysis using FTIR, XRD and solid-state ^31^P NMR

Throughout the discussion, we refer to each sample by the notation AlP_OH_(*T*), AlP_Cl_(*T*) or AlP_NO_3__(*T*), where the subscript identifies the precursor used in the synthesis and *T* denotes the annealing temperature. All the analysis was performed after cooling to room temperature.

**Fig. 1:**
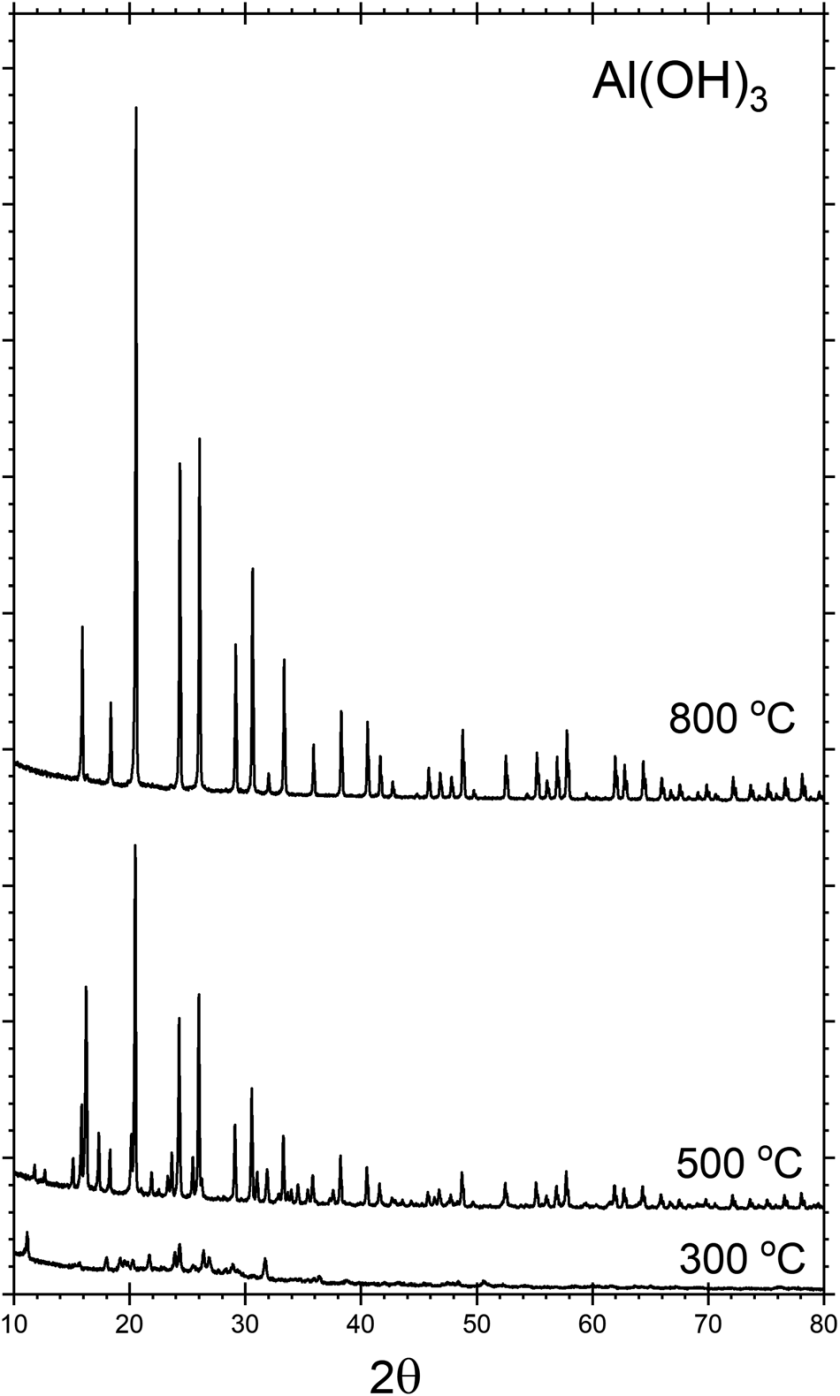
Powder XRD patterns recorded for the AlP_OH_ materials, with annealing at different temperatures. The AlP_OH_(300) sample is mostly non-crystalline, but both cubic metaphosphate and hexacyclophosphate are present in the AlP_OH_(500) sample. The AlP_OH_(800) sample is a pure phase of the cubic metaphosphate. Data for the AlP_Cl_ and AlP_NO_3__ materials are given in ESI.†

The crystalline phases present within each sample were investigated by powder XRD. Fig. 1, shows the XRD patterns for the AlP_OH_ samples. For AlP_OH_(300) [*i.e.*, the sample prepared from the Al(OH)_3_ precursor and annealed at 300 °C], only low intensity peaks are observed in the XRD data, and we have been unable to definitively match these peaks to a known structure. For the AlP_OH_(500) sample, the XRD data show sufficient crystallinity to allow Le Bail fitting, although Rietveld refinement was not possible. The Le Bail fitting confirms that two distinct phases are present: a cubic aluminium metaphosphate [Al(PO_3_)_3_] (ICSD: 26759) and an aluminium hexacyclophosphate (ICSD: 260723). For the AlP_OH_(800) sample, only the cubic aluminium metaphosphate is present. The results from Le Bail fitting are shown in Fig. S1 and S2 (in ESI†).

d'Yvoire^14,15^ also reported a pure cubic phase for a material prepared from Al(OH)_3_ and annealed at 800 °C, with a second phase present in the material annealed at 500 °C. However, d'Yvoire assigned the second phase to a monoclinic structure rather than the aluminium hexacyclophosphate observed here.

XRD indicates that the AlP_NO_3__(500) and AlP_NO_3__(800) samples (Fig. S3 and S4†) are a mixture of the cubic aluminium metaphosphate and aluminium hexacyclophosphate phases [similar to AlP_OH_(500) but different from AlP_OH_(800)]. Among the samples prepared from the AlCl_3_ precursor, the only crystalline product was AlP_Cl_(800), identified from XRD as pure cubic Al(PO_3_)_3_ (Fig. S5†).

**Fig. 2:**
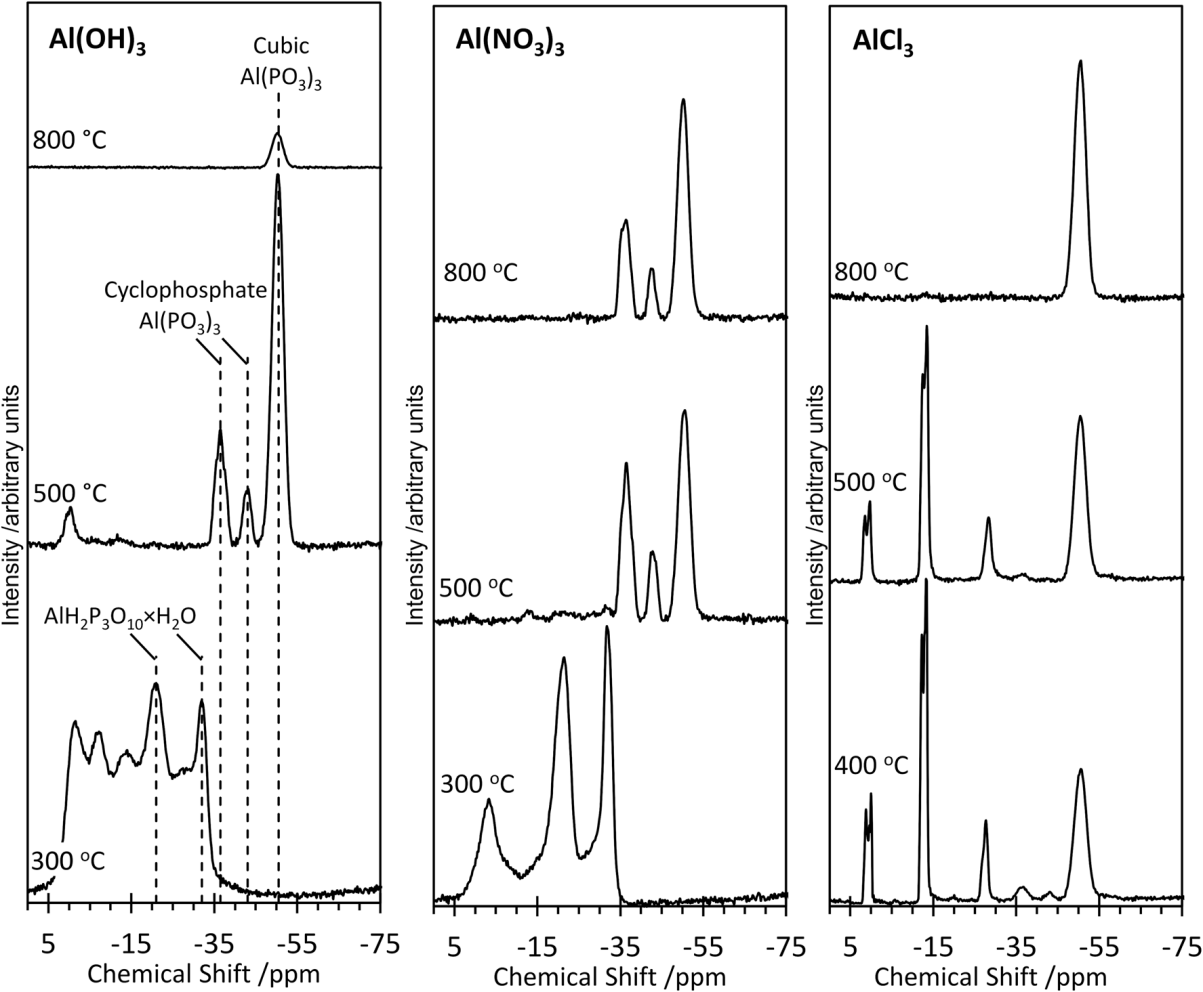
High-resolution solid-state ^31^P NMR spectra of the aluminium phosphate materials prepared from the Al(OH)_3_, Al(NO_3_)_3_ and AlCl_3_ precursors at different annealing temperatures. All spectra were recorded at room temperature.

**Fig. 3:**
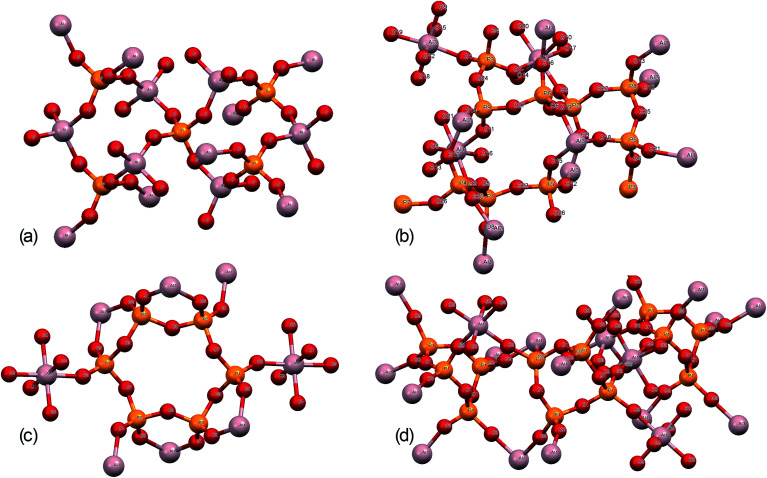
Structures of (a) aluminium orthophosphate (AlPO_4_), (b) monoclinic aluminium metaphosphate, (c) aluminium hexacyclophosphate, and (d) cubic aluminium metaphosphate.

Further structural insights are obtained from high-resolution solid-state ^31^P NMR spectra (Fig. 2). For several of the samples, a peak at 0 ppm is present and assigned as the phosphoric acid starting material. As the annealing temperature increases, there is a trend towards increasingly negative ^31^P chemical shifts, attributed to polymerization.^16^ For AlP_OH_(300), a broad set of overlapping peaks is observed between 5 ppm and −40 ppm, possibly suggesting an amorphous structure. The peaks at −21 ppm and −32 ppm (which represent *ca.* 20% of the total signal) are assigned^16^ to aluminium tripolyphosphate (AlH_2_P_3_O_10_·H_2_O). These peaks are also observed for AlP_NO_3__(300). For AlP_OH_(800), only one peak is observed (at 50.5 ppm) and is attributed unambiguously to cubic Al(PO_3_)_3_, consistent with the presence of a single phosphorus environment in this structure (Fig. 3). This peak is also present for the AlP_OH_(500) sample, together with peaks at −36.5 ppm and −43.0 ppm; the area ratio for these two peaks is 2 : 1, consistent with the presence of three crystallographically distinct phosphorus environments in the aluminium hexacyclophosphate phase (Fig. 3). Monoclinic Al(PO_3_)_3_, on the other hand, has 9 distinct phosphorus environments (Fig. 3).

For the AlP_NO_3__(300) sample, three peaks are observed between 5 ppm and −35 ppm [in contrast to the overlapping set of peaks observed in this region for AlP_OH_(300)], including peaks at −21 ppm and −32 ppm assigned to aluminium tripolyphosphate (AlH_2_P_3_O_10_·H_2_O), which represents ∼50% of the signal. The peak at −3.4 ppm is attributed to some remaining aluminium orthophosphate. For the AlP_NO_3__(500) sample, the ^31^P NMR spectrum contains peaks characteristic of the hexacyclophosphate (43.3%) and cubic metaphosphate (52%). Annealing at a higher temperature does not complete the transformation to the cubic metaphosphate as the ^31^P NMR spectrum for the AlP_NO_3__(800) sample clearly contains peaks due to the hexacyclophosphate phase (*ca.* 36% of the signal intensity).

The ^31^P NMR spectra for AlP_Cl_(400) and AlP_Cl_(500) are significantly different from those observed for the AlP_OH_ and AlP_NO_3__ materials. The major peaks are due to cubic metaphosphate (50.5 ppm), orthophosphate (1 ppm and 0 ppm), pyrophosphate (−12 ppm and −13.5 ppm) and polyphosphate (peaks in the range −20 ppm to −28 ppm), with only very weak peaks observed for hexacyclophosphate. In contrast, the AlP_Cl_(800) sample is a pure phase of cubic Al(PO_3_)_3_. These observations suggest that the metaphosphate formed from the AlCl_3_ precursor may be produced *via* a slightly different pathway than from the Al(OH)_3_ and Al(NO_3_)_3_ precursors. We deduce that the stability of the Al–Cl bond hinders formation of aluminium phosphate from the Al(Cl)_3_ precursor at lower temperatures, leaving the phosphoric acid to react mostly with itself to form varying degrees of polyphosphates. However, at the higher annealing temperature of 800 °C, the phosphate transforms completely to cubic Al(PO_3_)_3_.

### Surface analysis with XPS

**Fig. 4:**
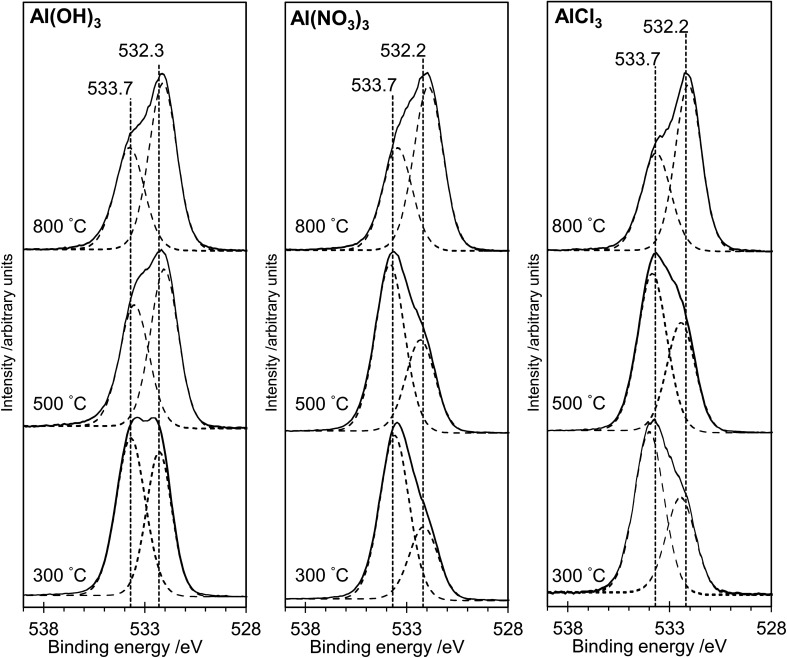
X-ray photoelectron spectra in the O(1s) region for the aluminium phosphate materials prepared from the Al(OH)_3_, Al(NO_3_)_3_ and AlCl_3_ precursors at different annealing temperatures. Solid lines show experimental data. The dotted curves show deconvolution using the methods described in the text. For all three precursors, the intensity of the peak at lower binding energy increases relative to the peak at higher binding energy as the annealing temperature is increased.

The XP spectra in Fig. 4 show the O(1s) data for all samples. For samples prepared from each of the three precursors, there is a general shift in peak position towards *lower* binding energy as the annealing temperature is increased. Curve fitting confirms that two distinguishable peaks are present at binding energies of *ca.* 533.7 eV and 532.3 eV in all spectra, with a transfer of intensity from the higher binding energy peak to the lower binding energy peak as the annealing temperature is increased (peak area ratios are given in Table 1). In particular, we note the close similarity between the spectra for the AlP_OH_(800), AlP_NO_3__(800) and AlP_Cl_(800) samples, all of which show an approximately 2 : 1 intensity ratio (lower : higher binding energy peaks) and resemble the O(1s) spectrum of aluminium metaphosphate published by Rotole and Sherwood.^12^

**Table 1:** Quantification of XP spectra

Reactant		Annealing temperature/°C					
		300/400		500		800	
Al(OH)_3_	O(1s) binding energy^a^	533.7	532.3	533.5	532.0	533.8	532.1
	% of peak area	55.5	44.5	44.6	55.4	39.0	61.0
	∼532 : 533 ratio	0.8		1.2		1.6	
	O : P ratio	3.2		2.9		3.0	
	P : Al ratio	5.3		5		3.8	
Al(NO_3_)_3_	O(1s) binding energy^a^	533.6	532.2	533.8	532.3	533.7	532.1
	% of peak area	69.1	30.9	64.4	35.6	38.5	61.5
	∼532 : 533 ratio	0.43		0.55		1.6	
	O : P ratio	2.8		3		2.9	
	P : Al ratio	n/a		10.2		3.6	
AlCl_3_	O(1s) binding energy^a^	534.0	532.5	533.9	532.4	533.7	532.1
	∼532 : 533 ratio	62.4	37.6	59.8	40.2	39.6	60.4
	Peak area ratio	0.6		0.7		1.5	
	O : P ratio	3.3		3.1		2.8	
	P : Al ratio	n/a		n/a		4.6	

a Binding energies are referenced to the C (1s) peak at 284.7 eV; curves are fitted with Gaussian–Lorentzian (GL (30)) line shapes.

**Fig. 5:**
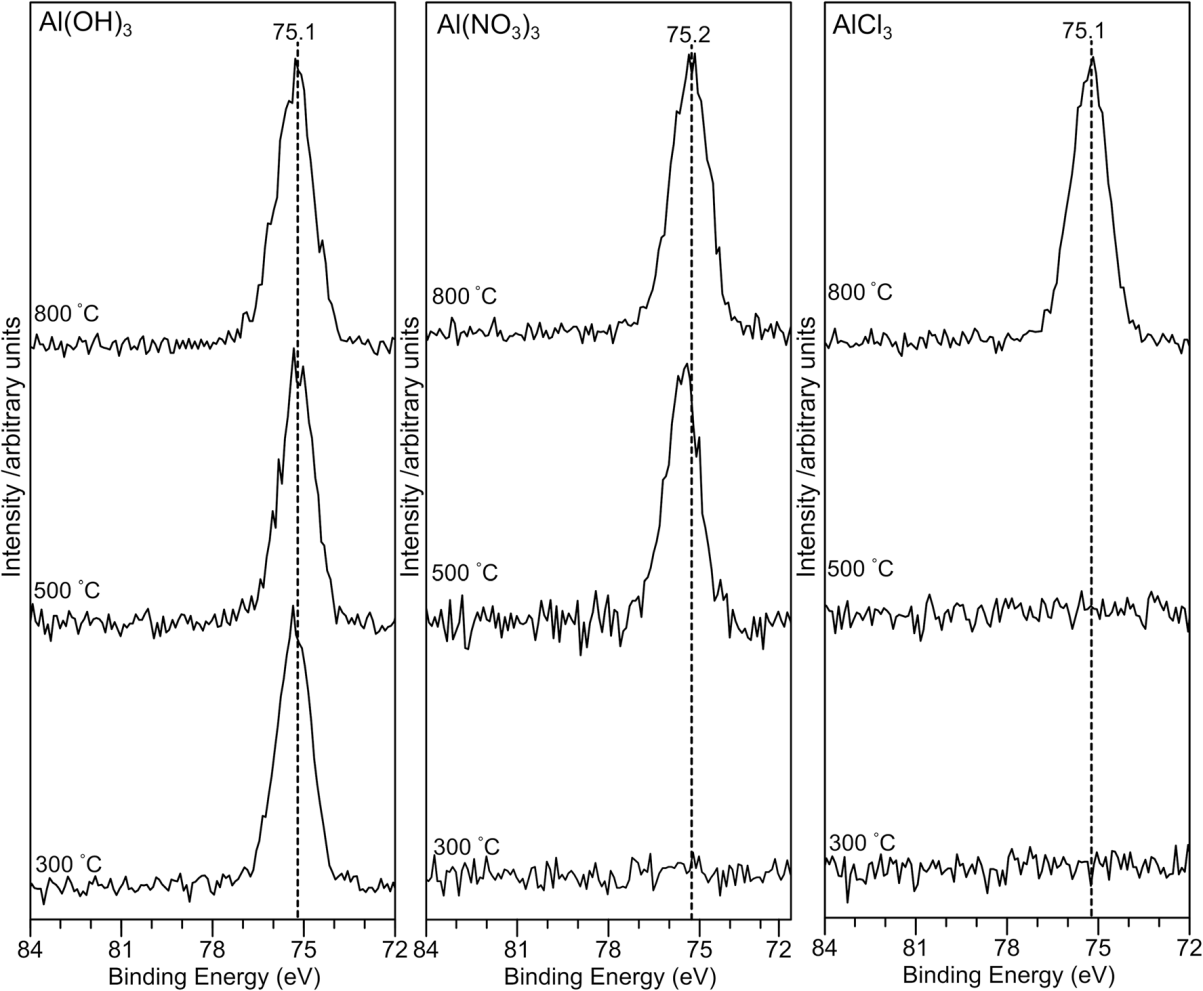
X-ray photoelectron spectra in the Al(2p) region for the aluminium phosphate materials prepared from the Al(OH)_3_, Al(NO_3_)_3_ and AlCl_3_ precursors at different annealing temperatures. The absence of any Al(2p) signal for lower annealing temperatures in the case of the Al(NO_3_)_3_ and AlCl_3_ precursors suggests that the surface is dominated by a hydrogen polyphosphate.

The Al(2p) XP spectra for all samples are shown in Fig. 5. For the AlP_OH_ samples, a strong peak is present for all annealing temperatures at ∼75.3 eV for AlP_OH_(300) and shifting slightly to 75.1 eV for AlP_OH_(800). However, for the AlP_NO_3__(300) sample, no peaks are observed in the Al(2p) XP spectrum, while a peak at *ca.* 75 eV is present for both AlP_NO_3__(500) and AlP_NO_3__(800). For the AlP_Cl_ samples, again no peaks are observed in the Al(2p) XP spectra for the AlP_Cl_(300) and AlP_Cl_(500) samples, while a peak is observed at *ca.* 75 eV for AlP_Cl_(800). This difference in behaviour parallels the physical appearance of the AlP_Cl_ samples, for which both AlP_Cl_(300) and AlP_Cl_(500) are tacky but AlP_Cl_(800) is not.

**Fig. 6:**
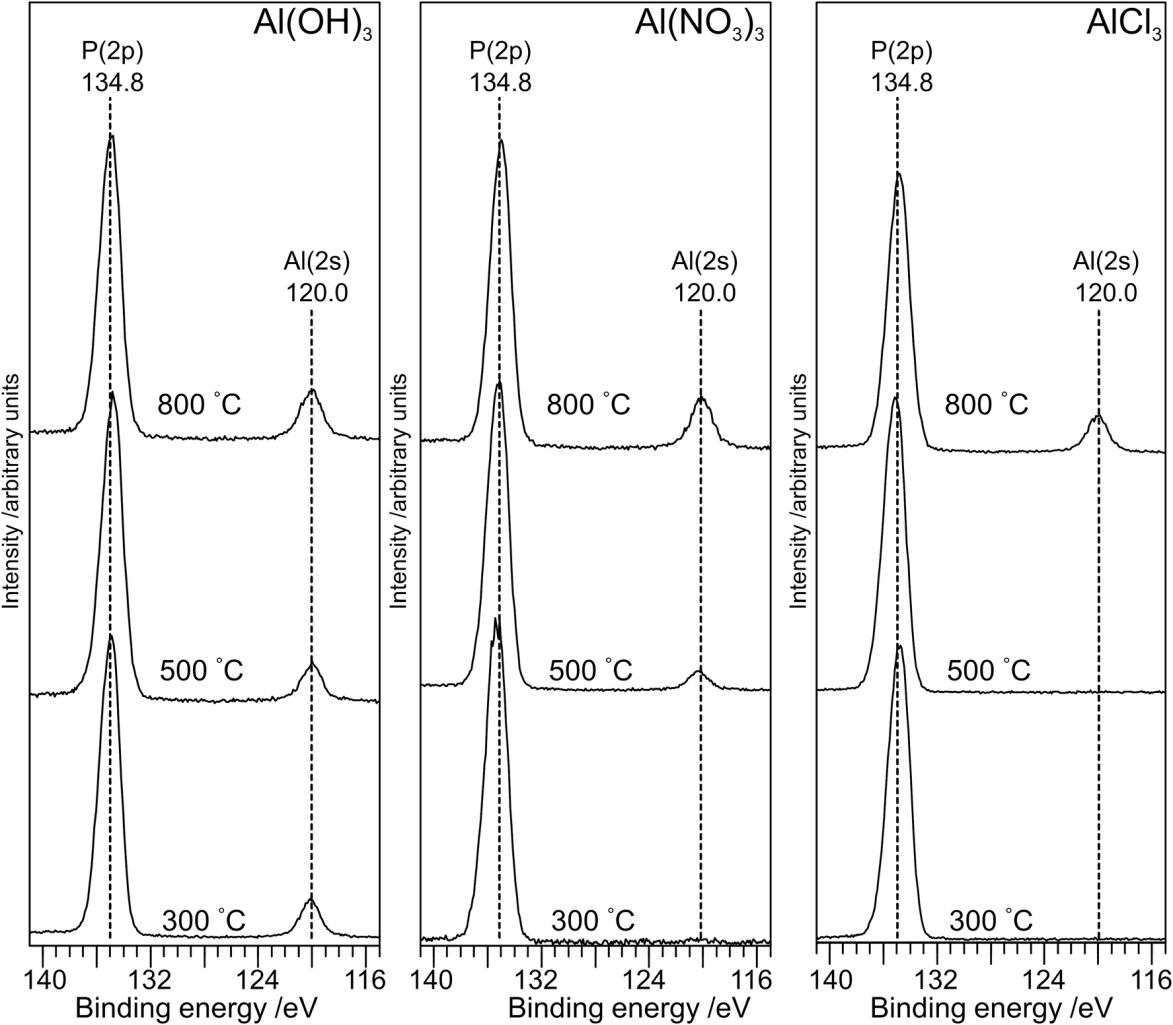
X-ray photoelectron spectra in the P(2p) and Al(2s) region for the aluminium phosphate materials prepared from the Al(OH)_3_, Al(NO_3_)_3_ and AlCl_3_ precursors at different annealing temperatures. For all three precursors, a small shift (∼0.2 eV) to lower binding is observed on annealing at 800 °C.

The XP spectra in the P(2p) region (Fig. 6) have a single peak at ∼134.8 eV for all samples with a small shift (∼0.2 eV) to lower binding energy as the annealing temperature is increased to 800 °C. The observed peak is consistent with the average peak position for metaphosphates in the NIST database^17^ (134.8 eV; *σ* = 0.5 eV) and with results of Rotole and Sherwood^10,11^ on aluminium phosphates. However, it is in marked contrast to sodium phosphates,^8^ for which the P(2p) binding energy shifts by 2 eV from the orthophosphate (132.5 eV) to the oxygen-bridged metaphosphate (134.5 eV).

The atomic ratios calculated from the XPS spectra (Table 1) are informative. The P : O ratio is very close to 1 : 3 for all samples but the P : Al ratio is always higher than 3 : 1. The fact that the XPS survey scans for materials prepared at lower annealing temperatures (Fig. S6†) do not contain any signal for aluminium or for any other cation suggests that the surface is dominated by hydrogen phosphates, with the consistent O : P ratio of 3 : 1 indicating extensive polymerization at the surface. We also note that no XPS signal due to chlorine is observed for any of the samples (Fig. S7†). Following annealing at 800 °C, the samples from all precursors are highly crystalline, and the presence of the Al(2p) peak in the XP spectra suggests that the surface is now dominated by aluminium metaphosphate. However, the P : Al ratio remains higher than the expected 3 : 1 ratio, particularly for AlCl_3_(800), suggesting that some hydrogen polyphosphates are present at the surface.

### Surface analysis with ATR-FTIR

**Fig. 7:**
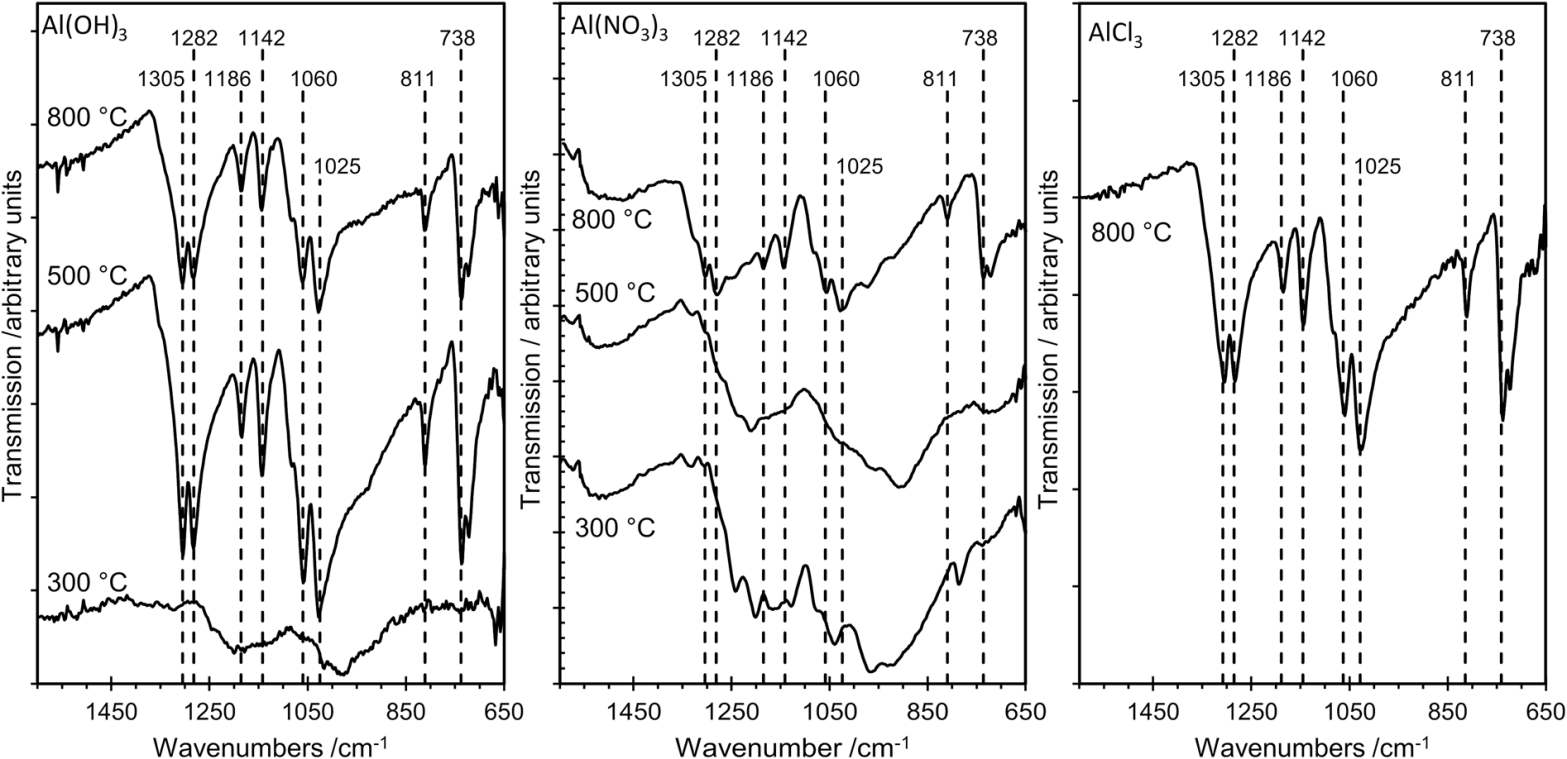
ATR-FTIR spectra of the aluminium phosphate materials prepared from the Al(OH)_3_, Al(NO_3_)_3_ and AlCl_3_ precursors at different annealing temperatures. At 300 °C the poorly resolved spectra are characteristic of aluminium orthophosphate, whereas aluminium metaphosphate dominates at 500 °C and 800 °C. The materials prepared from the AlCl_3_ precursor did not crystallize at the lower annealing temperatures, and ATR spectra were only recorded for the sample annealed at 800 °C.

The sampling depth of ATR-FTIR spectroscopy, typically between 0.5–2 μm, is significantly larger than that for XPS, which detects only the top 2–4 nm of the surface for the elements studied here. Nevertheless, there is excellent agreement between the IR results and the results from the more surface sensitive XPS method, as illustrated in Fig. 7, which shows FTIR data for several samples. For AlP_OH_(300), broad, weak bands are present in the range *ca.* 1100–1250 cm^−1^, characteristic of an aluminium orthophosphate with some indication of P–O–P bond formation from the weak peak at 1025 cm^−1^.^18^ The spectra for AlP_OH_(500) and AlP_OH_(800) are dominated by strong bands assigned to metaphosphates. In particular, the peak at 738 cm^−1^ is assigned to Al–O–P, bands at 811, 1025, 1060 and 1070 cm^−1^ are assigned to P–O–P modes, and bands at 1282 and 1305 cm^−1^ are assigned to P=O bonds in the aluminium metaphosphate.

## Discussion

The structures of aluminium orthophosphate, aluminium hexacyclophosphate and two aluminium metaphosphates are shown in Fig. 3, and can be used to rationalize the changes in the XP spectra observed for different annealing temperatures. Small shifts to lower binding energy are observed for the P(2p) and Al(2p) peaks as the annealing temperature is increased, but the most significant changes arise in the O(1s) spectra, which show a transfer of intensity from a higher binding energy peak at 533.7 eV to a lower binding energy peak at 532.3 eV. The best starting point to understand these changes is the O(1s) spectra for all samples annealed at 800 °C, as these spectra are all very similar to those reported by several other authors, including Gresch *et al.* for sodium metaphosphates,^8^ Crobu *et al.* for zinc phosphates,^9^ and Rotole and Sherwood for model aluminium phosphates.^11,12^ The FTIR, XRD and solid-state ^31^P NMR data indicate the presence of mainly cubic aluminium metaphosphate after annealing at 800 °C and we can therefore definitively assign the peaks in the O(1s) region at 533.7 eV and 532.3 eV, respectively, to oxygen atoms bridging between phosphorus atoms (P–O–P) and oxygen atoms bridging between phosphorus and aluminium atoms (P–O–Al), in agreement with Gresch *et al.*

In the metaphosphate, these two bonding environments are expected to be present in a 2 : 1 ratio of P–O–Al to P–O–P. However, as shown in Table 1, quantification of the XPS data for the samples annealed at 800 °C gives a peak area ratio (532.3 eV : 533.7 eV) of *ca.* 1.6 : 1, whereas the expected ratio is 2 : 1. Thus, the P : Al ratio at the surface of these materials is higher than the expected 3 : 1 ratio. To understand these differences, we now consider the XP spectra recorded for samples annealed to lower temperatures.

A key observation is that the AlP_NO_3__(300) and AlP_Cl_(300) samples show no evidence, in the XP spectra, for the presence of aluminium. The AlP_NO_3__(500) sample does show evidence for aluminium, but the AlP_Cl_(500) sample does not. The absence of aluminium indicates a purely hydrogen terminated phosphate material at the surface. Unreacted phosphoric acid can be ruled out based on the O : P ratio of 3 : 1, but there is evidence from the solid-state ^31^P NMR results for hydrogen terminated or cyclic polyphosphates (giving peaks at −28 ppm and −32 ppm) which would have a 3 : 1 ratio. A cyclic polyphosphate such as P_4_O_10_ has a P–O–P to P=O bond ratio of 1.5 : 1, which could account for the XPS ratios if the oxygen in P=O has a binding energy of ∼532 eV, overlapping with the XPS peak for the oxygen in P–O–Al. This assignment would be in agreement with Gresch *et al.*^8^ Finally, the “wet” physical appearance of samples annealed at lower temperatures is also consistent with the presence of hydrogen polyphosphates which would be poorly crystalline.

From the data presently available, we cannot determine whether annealing ultimately leads to sublimation or decomposition of the polymeric phosphates, or whether further reaction with unreacted aluminium precursor occurs. However, for samples annealed at 800 °C, the XP spectra are consistent with the presence of aluminium metaphosphates although the slightly higher P : Al ratio in the case of the material prepared from AlCl_3_ suggests the surface contains some hydrogen terminated polyphosphates.

## Conclusions

Aluminium metaphosphate is formed from the reaction of phosphoric acid with three different aluminium compounds (Al(OH)_3_, Al(NO_3_)_3_ and AlCl_3_) followed by annealing in air. XRD, XPS and FTIR measurements of the resulting materials show almost identical behaviour from all three precursors, but the solid-state ^31^P NMR spectra are significantly different at the lower annealing temperatures (300 °C and 500 °C). The unique solid-state ^31^P NMR spectra of the materials annealed at lower temperatures indicates the presence of amorphous materials which would not be identified by XRD, but explains the lack of an Al(2p) signal in XP spectra of the materials prepared from the Al(NO_3_)_3_ and AlCl_3_ precursors at lower annealing temperatures. For all three precursors, a cubic metaphosphate is produced on annealing at 800 °C, with a hexacyclophosphate present at lower annealing temperatures in the case of the Al(NO_3_)_3_ and Al(OH)_3_ precursors. The XP spectra in the O(1s) region of the aluminium phosphate materials show two components at 532.3 eV and 533.7 eV, which are definitively assigned to the P–O–Al and P–O–P bonding environments, respectively. However, samples annealed at lower temperatures also exhibit surface species assigned as cyclic polyphosphates, with binding energies of 532.3 eV and 533.7 eV for the P=O and P–O–P bonding environments, respectively.

## Conflicts of interest

There are no conflicts to declare.

## Supplementary Material

RA-010-C9RA08738A-s001
