# Supplementary material for: Rationalization of the X-ray photoelectron spectroscopy of aluminium phosphates synthesized from different precursors
Source: RSC Adv. 2020 Feb 26;10(14):8444–52. doi: 10.1039/c9ra08738a (PMC9050013; doi:10.1039/c9ra08738a)
Supplement: RA-010-C9RA08738A-s001 [file RA-010-C9RA08738A-s001.pdf]

Electronic Supplementary Material (ESI) for RSC Advances.
This journal is © The Royal Society of Chemistry 2020

Supplementary Information

Rationalization of the XP Spectroscopy of Aluminium Phosphates Synthesized from Different Precursors

Victoria Bemmer[c], Michael Bowker[a], James H. Carter[a], Philip R. Davies[a]*, Lee E. Edwards[a], Kenneth D. M. Harris[b], Colan E. Hughes[b], Fiona Robinson[d], David J.Morgan[a], Matthew G. Thomas[a]

[a.] *Cardiff Catalysis Institute, School of Chemistry, Cardiff University, Cardiff, CF10 3AT, UK.*
[b.] *School of Chemistry, Cardiff University, Cardiff, CF10 3AT, UK.*
[c.] *Current address: Dept. of Materials, Imperial College, South Kensington Campus, London SW7 2AZ, UK.*
[d.] *Cogent Power Ltd, Newport NP19 0RB, UK*

Powder XRD Patterns and Le Bail fitting

Red crosses, experimental data points; green line, calculated data; magenta line, difference plot; black tick marks, predicted peak positions for cubic metaphosphate; cyan tick marks; predicted peak positions for hexacyclophosphate.

Materials synthesized from the aluminium hydroxide precursor

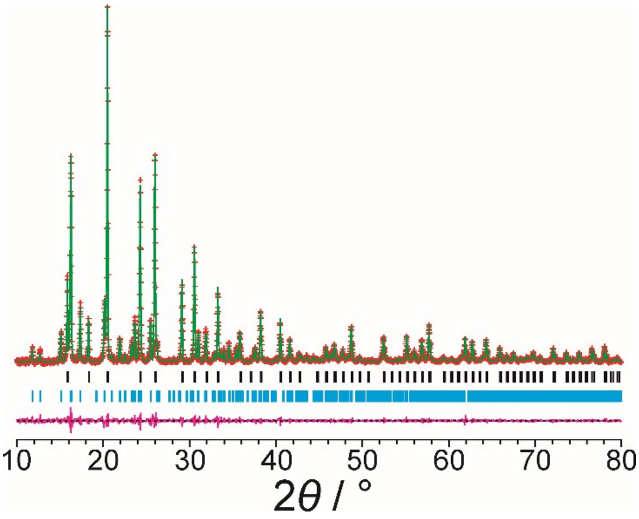

Figure S1: Le Bail fitting for AlP_{OH}(500), confirming that both cubic aluminium metaphosphate (black lines) and aluminium hexacyclophosphate (cyan lines) are present.

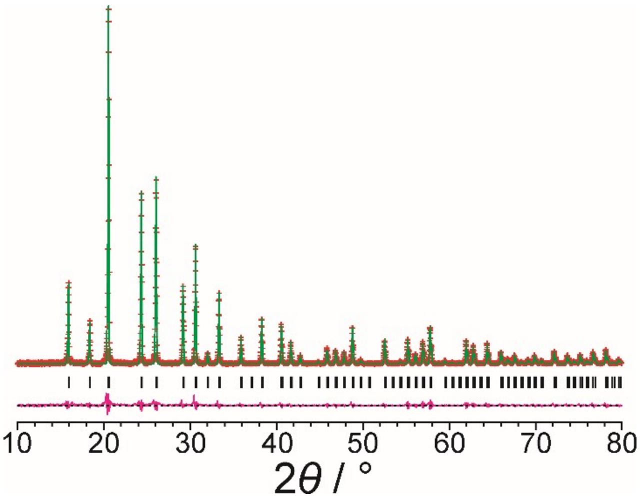

Figure S2: Le Bail fitting for AlP_{OH}(800), confirming that cubic aluminium metaphosphate is the only phase present.

Materials synthesized from the aluminium nitrate precursor

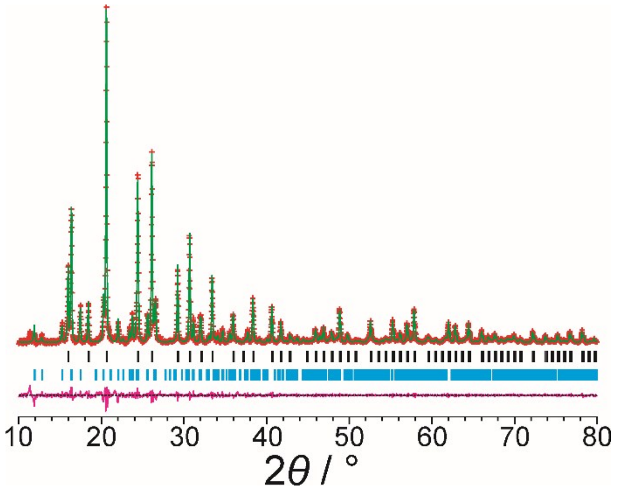

Figure S3: Le Bail fitting for AlP_{NO3}(500), confirming that

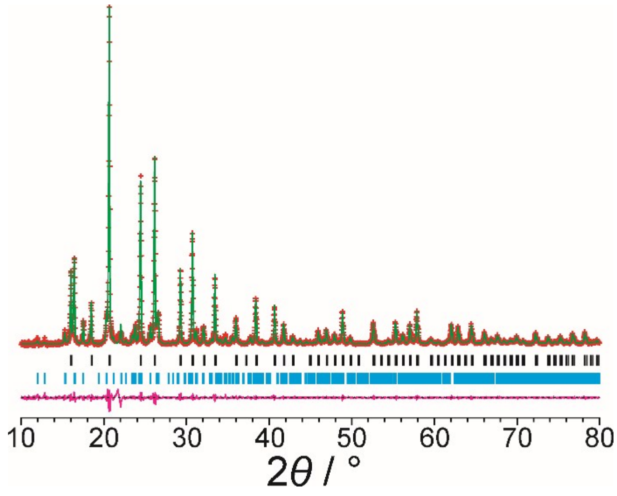

Figure S4: Le Bail fitting for AlP_{NO3}(800), showing that

both cubic aluminium metaphosphate (black lines) and aluminium hexacyclophosphate (cyan lines) are present. There is also evidence that a third phase is present, which we have been unable to identify.

both cubic aluminium metaphosphate (black lines) and aluminium hexacyclophosphate (cyan lines) are present at this temperature.

Materials synthesized from the aluminium chloride precursor

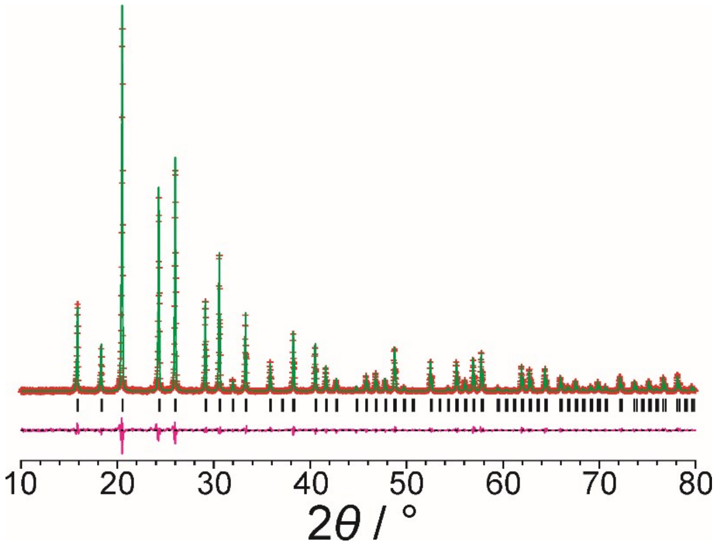

Figure S5: Le Bail fitting for AlP_{Cl}(800), confirming that cubic aluminium metaphosphate is the only phase present.

XP Spectra

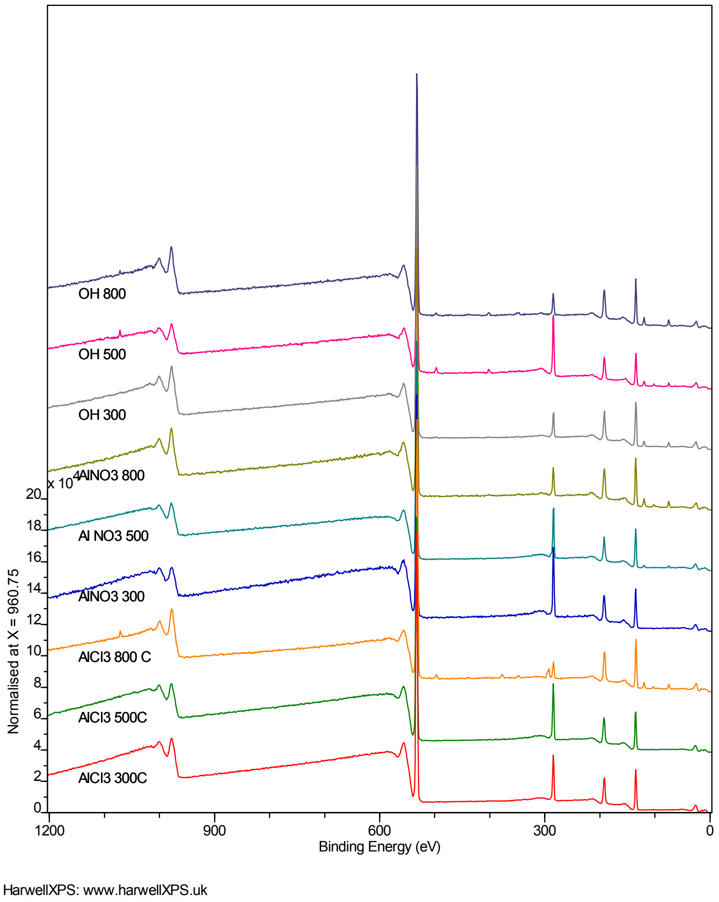

Figure S6: XP widescan for all samples prepared from different

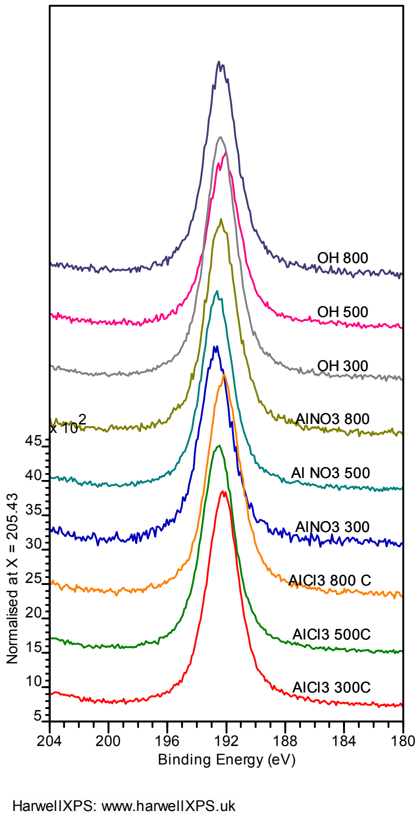

Figure S7: Cl(2p) region of the XP spectrum. The peak at 192 eV corresponds to the P(2s) peak of an oxidised phosphorus.

precursors and at different annealing temperatures.
